# Supplementary material for: Transition to adult care: Exploring factors associated with transition readiness among adolescents and young people in adolescent ART clinics in Uganda
Source: PLoS One. 2021 Apr 29;16(4):e0249971. doi: 10.1371/journal.pone.0249971 (PMC8084193; doi:10.1371/journal.pone.0249971)
Supplement: S1 File — (DOCX) [file pone.0249971.s002.docx]

## ANNEX 3: ASSESSEMENT TOOL FOR READINESS TO TRANSITION MEDICAL CARE

**Names: ________________________________ Gender: M/ F ID #:__________**

**DOB: ____/_____/_______ Assessment date**: ____________________________

**Checklist for Successful Transition**

***Please ensure the following are assessed at least annually to enable a successful transition***

- *Client knows about his/her illness and oriented towards future goals and hopes, including long-term survival.*
- *Client has skills needed to negotiate appointments and multiple providers in an adult practice setting.*
- *Client has personal and medical independence and is able to assume responsibility for his/ her treatment and participate in decision-making.*
- *Client is active in and has been receiving uninterrupted care.*
- *Client’s basic and psychosocial needs, such as housing, employment, education, home-based services, or transportation have also been addressed.*
- *Client is familiar with the new providers and setting, and has participated in discussions of the transition plan for self.*

**INSTRUCTIONS: PLEASE CIRCLE ONE RESPONSE PER QUESTION:**

**A] KNOWLEDGE OF OWN HEALTH:**

1. Does the adolescent know and understand the cause of his/her medical condition?

**Yes          No          N/A**

1. Does the adolescent know and understand the changes related to his/her medical condition?

**Yes          No          N/A**

1. Does the adolescent know the daily medications he/she takes?

**Yes          No          N/A**

1. Does the adolescent know/ understand what the medications he/she takes do?

**Yes          No          N/A**

1. Does the adolescent have difficulties with his/her daily medications?

**Yes          No          N/A**

**If yes, what are they?
 ________________________________________________________________**

**________________________________________________________________**

1. Does the adolescent know the current results of his/her :**Viral load: _______________________________**

**B] KNOWLEDGE OF RESPONSIBLE BEHAVIOR (What to do to keep healthy)**

1. Does the adolescent drink any form of alcohol, tobacco or other drugs?

**Yes          No          N/A**

*Specify: ______________________________________________*

1. Is the adolescent engaging in unprotected sex?

**Yes          No          N/A**

*Specify: ______________________________________________*

1. Does the adolescent exercise regularly?

**Yes          No          N/A**

*Specify: ______________________________________________*

1. Does the adolescent take his / her medications independently?

**Yes          No          N/A**

**5. Does the adolescent appreciate and follow a balanced diet for a health living**

**C] KNOWLEDGE OF RESPONSE TO EMERGENCY CARE:**

1. Does the adolescent have a phone to use in case of an emergency?

**Yes          No          N/A**

*If yes, what is the telephone number _________________________*

2. Does the adolescent have a telephone number(s) of close friends, relative and or family to call in case of an emergency?

**Yes          No          N/A**

*If yes, nature of person and the telephone number___________________________________*

3. Does the adolescent know where the closest health unit/ center/ hospital is located when he/she need to see a doctor?

**Yes          No          N/A**

***If yes, specify the name ______________________________________________***

4. Is the adolescent able to use public transportation when in need of it to go for care?

**Yes          No          N/A**

**D] KNOWLEDGE OF HOW TO MANAGE HEALTHCARE NEEDS:**

1. Is the adolescent able to decide and agree on appointments with healthcare providers?

**Yes          No          N/A**

1. Does the adolescent have a person to support him/her at home or escort to clinic?

**Yes          No          N/A**

1. Does the adolescent have an attendant or buddy at home?

**Yes          No          N/A**

1. Is the adolescent responsible for his/her supervision and upkeep?

**Yes          No          N/A**

1. Does the adolescent know how to seek for care and support for health related concerns? **Yes No N/A**
2. Is the adolescent able to use a bus, taxi and/or other types of public transportation? **Yes No N/A**

**E] DEMONSTRATION OF RESPONSIBLE SEXUAL BEHAVIOR:**

1. Does the adolescent understand what sexual relationships are and what is involved?

**Yes          No          N/A**

2. Does the adolescent know about contraception and ways to prevent STDs?

**Yes          No          N/A**

3. Does the adolescent know when and where to seek birth control counseling?

**Yes          No          N/A**

4. Does the adolescent understand the problems associated with unplanned pregnancies?

**Yes          No          N/A**

5. Does the adolescent understand the responsibilities of being a parent?

**Yes          No          N/A**

**F] KEEPING TRACK OF HEALTH NEEDS:**

1. Does the adolescent have a copy of his/her health records?

**Yes          No          N/A**

2. Does the adolescent keep track of his/her calendar health appointments?

**Yes          No          N/A**

**3. Is the adolescent** able to receive prescriptions and obtain refills of his/her medications?

**Yes No N/A**

4. Does the adolescent know the possible side effects of medications?

**Yes No N/A**

5. Is the adolescent able to identify members of the health care team, roles and how to contact them?

**Yes No N/A**

6. Does the adolescent carry phone contacts of friends and family when he/she travels?

**Yes          No          N/A**

**G] SUPPORT GROUPS:**

1. Is the adolescent enrolled in any HIV+ Support Group or any other support group?

**Yes          No**

2. Which are these support groups?

   Transition group; **Yes          No**

Adolescent Peer support groups; **Yes          No**

Teen Mother’s Group; **Yes          No**

Teen Father’s Group;**Yes No**

3. Does the adolescent interact directly with group members and support team?

**Yes No N/A**

| **H] TRANSITION PLAN:** |
| --- |
| Did the adolescent, care giver and health worker draw a transition plan?  Yes          No  Did the adolescent meet the adult team / clinician? (It is better for the first visit to be at the paediatric clinic)  Yes          No  Has the adolescent had any appointment with the adult clinic clinician? (It’s better for the adolescent to have first appointment while still at the paediatric clinic)  Yes          No |

**TRANSITION STATUS REPORT**

**Date: ____________________________**

**Total Score: __ /38** Transition if Score is >30/38

Consider transition after 3 months if score is 25 - 30/38

Initial adult care appointment: ____________________________

First appointments follow up plan: ______________________________________________________________________

Responsible person(s):

______________________________________________________________________
(Names of Transition Coordinator) (Signature of Transition Coordinator) (Date)

______________________________________________________________________
