# Supplementary material for: Transition to adult care: Exploring factors associated with transition readiness among adolescents and young people in adolescent ART clinics in Uganda
Source: PLoS One. 2021 Apr 29;16(4):e0249971. doi: 10.1371/journal.pone.0249971 (PMC8084193; doi:10.1371/journal.pone.0249971)
Supplement: Attachment — Submitted filename: Review-PONE-D-21-00972.docx [file pone.0249971.s003.docx]

Reviewer comments – PONE-D-21-00972

Manuscript title:

**Transition to adult care: Exploring factors associated with transition readiness among adolescents and young people in adolescent ART clinics in Uganda**

**Study Summary**

The study of Mbalinda and colleagues aims at exploring factors associated with adolescent readiness for the transition into adult care in Uganda. To achieve this goal, the authors conducted a cross-sectional study involving 786 individuals, aged 10 years to 24 years, among whom they explored the readiness for transition to adult clinics using structured interviews. The results indicated that 51 (6.5%) of the participants “adolescents” were ready to transition to adult care, based on the readiness to transition tolls they employed. The authors discussed their findings around this low readiness to transition in the ART program in Uganda.

**Reviewer observations**

Children and adolescent currently represent key populations with increasing challenges regarding HIV/AIDS care and monitoring management. It is obvious that specific strategies should be implemented for those key populations and should be adapted to their specific needs. Transition from pediatric services to adult wards is an important step, which may significantly affect the future outcome of ART of the adolescent if not adequately managed. This highlights the importance of such investigations that may help identifying factors that can affect the success of the transition.

**Major queries**

**A. The study population**

According to WHO, adolescents refer to individuals aged 10 to 19 years. This population is managed in pediatric/adolescent services until they reach the age for transition to adult services.

The study population of this report includes both adolescents and young adults aged from 10 to 24 years. It not clear in this study design and it is not indicated anywhere in the manuscript if the age group considered in Uganda for adolescent is 10-24 years. I believe not, since the authors consider the group from 20 to 24 years old as “young adults” and not adolescents.

In addition, in the methods presented, there is no indication if this study population “adolescent and young adults” was recruited in pediatric/adolescent services or in adult services as well. It is only mentioned (lines 120-121) that they were recruited “ …in antiretroviral therapy (ART) clinics in four regions of Uganda”.

This is a critical aspect of the validity of this study, since adequate definition of the population studied is essential to achieve the expected outcome. Is not clear how assessing transition readiness among a so wide age group (10 to 24) using the same investigation tools/questionnaires, will permit identifying factors affecting the transition. Are individuals aged 10, 11, 12, 13, 14 years, etc, expected to be ready for transition to adult services?

The high heterogeneity of this population can be the main reason of the very low transition readiness that the authors reported 51/786 (6.5%).

There is no rational on the selection of the different aged groups:

- What was expected as outcome from the population of 10-14? Is it expected that adolescent of this age group are ready for the transition?
- For those aged 20-24 years, are they still follow-up in pediatric/adolescent services? If no, why was this population included in the study?
- If the age of transition to adult services in Uganda is 24 years, in this case even for the group of 15-19 years, it will be hard to expect the same perception of readiness to transition compared to those aged 20-24 years.

This key aspect of the study, in my opinion, critically affects the validity of the results obtained.

**B. Other key points**

- Lines 80-88: the characteristics presented as referring to “transition readiness” are not supported by any reference. Are these definitions made by the authors, Ugandan authorities or international organizations? No reference presented.
- Lines 100-101: *“…Further, those at the highest risk for health complications and transmission of HIV to others (males, those living further away from health facilities) are…*”. Do authors have specific references to support this?
