# Supplementary material for: Transition to adult care: Exploring factors associated with transition readiness among adolescents and young people in adolescent ART clinics in Uganda
Source: PLoS One. 2021 Apr 29;16(4):e0249971. doi: 10.1371/journal.pone.0249971 (PMC8084193; doi:10.1371/journal.pone.0249971)
Supplement: Attachment — Submitted filename: Response to reviewers comments.docx [file pone.0249971.s004.docx]

**Makerere	University**

P.O. Box 7072 Kampala, Uganda	**Tel:** 256 414 530404

**E-mail**: [nursing@chs.mak.ac.ug](mailto:nursing@chs.mak.ac.ug)

**SCHOOL OF HEALTH SCIENCES**

**Department of Nursing**

22^nd^ February 2021

The Editor,

**PLOS ONE**

Dear Sir.

**Re: Response to Reviewers comments**

Thank you for your encouragement to revise and to resubmit our manuscript entitled: **"Transition to adult care: Exploring factors associated with transition readiness among adolescents and young people in adolescent ART clinics in Uganda."**

Attached is our resubmission.

We have attempted to address all the reviewers' comments and recommendations. We believe that our manuscript has been strengthened, and we thank the reviewers for taking the time to read the manuscript and to specify these recommendations

Yours faithfully,

***ScoviaNalugoMbalinda***

***Makerere University, College of Health Sciences, Department of Nursing***

***P.O. Box 7072, Kampala, Uganda***

***E-mail:*** [smbalinda@gmail.com](mailto:smbalinda@gmail.com).

**Comments**

**Major queries**

**A. The study population**

1. According to WHO, adolescents refer to individuals aged 10 to 19 years. This population is managed in pediatric/adolescent services until they reach the age for the transition to adult services.

**Response: Yes, adolescents are aged 10-19, but when we went to ART clinics, there were young people (aged above 19 years) who were still attending the clinic for HIV care services. Secondly, some adolescents are aged 19 years, and less attend HIV care in the adult HIV clinic.**

2. The study population of this report includes both adolescents and young adults aged from 10 to 24 years. It not clear in this study design and it is not indicated anywhere in the manuscript if the age group considered in Uganda for adolescent is 10-24 years. I believe not, since the authors consider the group from 20 to 24 years old as "young adults" and not adolescents.

**Response: Adolescents are aged 10-19, but we found that the adolescents had refused to go to adult ART clinics. We included adolescents (10-19 years) and young people to capture the age group 20-24. That why we included young people in the topic and the methodology we have stated that adolescents and young people. Page line 126,130 on page 6.**

**The provision of HIV care in this period is a continuum. The anomaly is that some adolescents attend HIV care in adult clinics while some young people still seek HIV care in adolescent clinics. Both scenarios potentially affect the quality of care received. That's why in the topic, we included adolescents and young people in the study to analyse the transition of HIV care from adolescent to adult clinics, with a focus on transition readiness from adolescent to adult HIV care. The questions where information was needed are whether, irrespective of age, the population of adolescents and young people were ready to transition from adolescent to adult clinics, as were their readiness for the transition process. Line 113-116, page 6.**

3. In addition, in the methods presented, there is no indication if this study population "adolescent and young adults" was recruited in pediatric/adolescent services or in adult services as well. It is only mentioned (lines 120-121) that they were recruited "…in antiretroviral therapy (ART) clinics in four regions of Uganda".

**Response: The adolescents and young people were recruited from adolescent ART clinics in each facility. The adolescent clinics were running once a week. Line 130, Page 6.**

**The participants' selection was the age of the adolescents and young people who were attending HIV care in the adolescent clinics.**

4. This is a critical aspect of the validity of this study since an adequate definition of the population studied is essential to achieve the expected outcome. It is not clear how assessing transition readiness among a so wide age group (10 to 24) using the same investigation tools/questionnaires will identify factors affecting the transition. Are individuals aged 10, 11, 12, 13, 14 years, etc, expected to be ready for transition to adult services?

**Response: The preparation of transitioning starts early as per standard operating procedures.**

**It starts 10-13 years by making transition plans and addressing concerns about support for parents/ care during transitions. As adolescents grow, they add other issues of independence and responsibility for themselves. Every 3-6 months, the adolescents are assessed as per the protocol described in the paper. If they score 30 and above, then they are ready to transition.**

**The age of transition: Most HIV- infected adolescents transition to adult care between 22 and 24 years of age. However, developmental stages and readiness for transition may be better indicators than chronological age for determining when the transition should occur. Patients with developmental delays or chaotic and unstable life may need more time to become ready to transition. Adolescents who demonstrate independence in making their own decision and show responsibility for their care may be ready sooner.**

**We had	earlier explained the process in line 104-112, page 5-6**

5. The high heterogeneity of this population can be the main reason of the very low transition readiness that the authors reported 51/786 (6.5%).

**Response: Yes, that could be the reason, however like we said, the transition is a process, and it starts as early as ten years, and the adolescents are assessed on different aspects like HIV knowledge and disclosure, Transition process, Knowledge of own health, Knowledge of responsible behavior, Knowledge of response of emergency care, Knowledge of how to manage health care needs, Demonstration of responsible sexual behavior, keeping track of health needs, support groups, and transition plan. Each adolescent is assessed in an individualized manner for skills development and understanding of successful transition and Address barriers for each patient that may prevent skills acquisition and providers from developing a transition plan and then implementing that plan.**

6. There is no rational on the selection of the different aged groups:

- What was expected as outcome from the population of 10-14? Is it expected that adolescent of this age group are ready for the transition?

**Response: Transition process is a process. It does not start at the age of transition but as early as ten years. So irrespective of the age as long you are ready for the transition as per protocol you will be transitioned.**

- For those aged 20-24 years, are they still follow-up in pediatric/adolescent services? If no, why was this population included in the study?

**Response: Yes, this group was in the adolescent clinic, and they had failed to transition. That is why they were included in the study**

- If the age of transition to adult services in Uganda is 24 years, in this case even for the group of 15-19 years, it will be hard to expect the same perception of readiness to transition compared to those aged 20-24 years.

**Response: Initially, the transition age was 19. However, the Ministry of health realized that some of the adolescents were not ready for the transition. It increased the age of transition to adult care between 22-24 years of age. But protocol stated that developmental stages and readiness for transition might be better indicators than chronological age for determining when the transition should occur. Patients with developmental delays or chaotic and unstable life may need more time to become ready to transition. Adolescents who demonstrate independence in making their own decision and show responsibility for their own care would transition earlier.**

**B. Other key points**

- Lines 80-88: the characteristics presented as referring to "transition readiness" are not supported by any reference. Are these definitions made by the authors, Ugandan authorities, or international organizations? No reference presented.

**Response: Reference is added. Line 88, page 5**

- Lines 100-101: *"…Further, those at the highest risk for health complications and transmission of HIV to others (males, those living further away from health facilities) are…*". Do authors have specific references to support this?

**Response: The references are there 11-13**

Journal Requirements:

When submitting your revision, we need you to address these additional requirements.

1) Please ensure that your manuscript meets PLOS ONE's style requirements, including those for file naming. The PLOS ONE style templates can be found at

<https://journals.plos.org/plosone/s/file?id=wjVg/PLOSOne_formatting_sample_main_body.pdf> and

<https://journals.plos.org/plosone/s/file?id=ba62/PLOSOne_formatting_sample_title_authors_affiliations.pdf>

**Response: This has been addressed in the manuscript**

2) In the Methods section, please provide a justification for the stratification of readiness to transition score used in the study (ie, please provide a justification as to why scores of 30< were classified as ready to be transitioned).

**Response: The score was used because it's a score that the Ministry of Health uses as a cut-off to show whether the adolescent or young person is ready for the transition. The total score of the tool was 38. If the young person scored 30 and above, they were ready to be transitioned. If they scored 25- 30, they would be reassessed and transitioned after three months in case they score 30. I have added the reference for this line 158, page 8. I have also attached the tool as a supporting file.**

3) Please include additional information regarding the survey or questionnaire used in the study and ensure that you have provided sufficient details that others could replicate the analyses. For instance, if you developed a questionnaire as part of this study and it is not under a copyright more restrictive than CC-BY, please include a copy, in both the original language and English, as Supporting information.

**Response: Ministry of Health developed the tool to aid the transition in the clinical area. We only added the socio-demographic and clinical, and immunological data. We have attached it as a supporting file**

4) We suggest you thoroughly copyedit your manuscript for language usage, spelling, and grammar. If you do not know anyone who can help you do this, you may wish to consider employing a professional scientific editing service.

**Response: We have edited the manuscript**

Upon resubmission, please provide the following:

- The name of the colleague or the details of the professional service that edited your manuscript
- A copy of your manuscript showing your changes by either highlighting them or using track changes (uploaded as a *supporting information* file)
- A clean copy of the edited manuscript (uploaded as the new *manuscript* file)

**Response: This has been addressed**

5)  We note that the grant information you provided in the 'Funding Information' and 'Financial Disclosure' sections do not match.

When you resubmit, please ensure that you provide the correct grant numbers for the awards you received for your study in the 'Funding Information' section.

**Response: This has been addressed**

6) Please amend your list of authors on the manuscript to ensure that each author is linked to an affiliation. Authors' affiliations should reflect the institution where the work was done (if authors moved subsequently, you can also list the new affiliation stating "current affiliation:…." as necessary).

**Response: This has been addressed**

7) Please include captions for your Supporting Information files at the end of your manuscript, and update any in-text citations to match accordingly. Please see our Supporting Information guidelines for more information: <http://journals.plos.org/plosone/s/supporting-information>.

**Response: This has been addressed Page line 555, page 30**
